# Sampling insulin in different tissue compartments using microdialysis: methodological aspects

**DOI:** 10.1038/s41598-020-78728-x

**Published:** 2020-12-15

**Authors:** Alexandra Högstedt, Bijar Ghafouri, Erik Tesselaar, Simon Farnebo

**Affiliations:** 1 Department of Surgery in Linköping, and Department of Biomedical and Clinical Sciences, Faculty of Health Sciences, Linköping University, 58185 Linköping, Sweden; 2 Pain and Rehabilitation Centre, and Department of Health, Medicine and Caring Sciences, Linköping University, Linköping, Sweden; 3 Department of Medical Radiation Physics, and Department of Health, Medicine and Caring Sciences, Linköping University, Linköping, Sweden; 4 Department of Hand Surgery, Plastic Surgery and Burns, and Department of Biomedical and Clinical Sciences, Linköping University, Linköping, Sweden

**Keywords:** Blood flow, Skin models

## Abstract

Sampling the concentration of insulin in human skin using microdialysis is challenging because of low intracutaneous concentrations and low recovery, presumably due to adsorption of insulin to the microdialysis system. In this study, we aimed to (1) measure how the concentration of insulin varies in three different tissue compartments (intracutaneous, subcutaneous and intravenous) and (2) to study how much insulin is adsorbed to the microdialysis catheter membranes and tubing during a typical microdialysis experiment, both in vivo and in vitro. We hypothesized that (1) the concentration of insulin decreases from the intravenous compartment to the intracutaneous and subcutaneous tissue, and that (2) adsorption of insulin to the microdialysis membrane and tubing impairs the recovery of insulin from the tissue. In this experimental study, microdialysis catheters were inserted intracutaneously, subcutaneously and intravenously in 11 healthy subjects. Systemic endogenous hyperinsulinemia was induced by intake of an oral glucose load. Insulin concentration was measured in the dialysate and in the extracted samples from the catheter membrane and tubings. In vitro microdialysis was performed to investigate the temporal resolution of the adsorption. After an oral glucose load insulin concentration increased intravenously, but not in the intracutaneous or subcutaneous compartments, while glucose, lactate and pyruvate concentrations increased in all compartments. The adsorption of insulin to the microdialysis membrane in vivo was highest in the intravenous compartment (*p* = 0.01), compared to the intracutaneous and subcutaneous compartments. In vitro, the adsorption to the microdialysis membrane was highest one hour after sampling, then the concentration gradually decreased after three and five hours of sampling. The concentration of insulin in peripheral tissues is low, probably due to decreasing tissue vascularity. Adsorption of insulin to the microdialysis membrane is modest but time-dependent. This finding highlights the importance of a stabilization time for the microdialysis system before sampling tissue analytes.

## Introduction

The relationship between the metabolic effects of insulin and its effects on local microcirculation is not fully understood. It is well known that there is a relationship, however not fully understood, between metabolism and microcirculation in the human body, primarily through the effects of the hormone insulin^1–3^. Insulin increases glucose metabolism and causes vasodilation through the endothelial nitric oxide pathway, boosting its own delivery to the peripheral tissue^2,3^. Microdialysis is a method that enables monitoring of concentrations of specific substances, changes in local metabolic conditions in the interstitium of a tissue, and alterations in local blood flow^4,5^. Therefore, microdialysis is a suitable technique for studying both the local concentration of insulin, while at the same time monitoring metabolic as well as microvascular effects after ingestion of carbohydrates.

These effects have previously been investigated in adipose tissue and skeletal muscle^6^ and recently in the skin^2,3^. Sampling insulin in the human skin with microdialysis technique has however appeared to be challenging in previous studies, because the concentration of insulin is believed to be much lower in less perfused tissues compared to in blood and muscle^2,3^. Another challenge is that large molecules are difficult to recover from the tissue using microdialysis. Previous studies have suggested that the recovery may be as low as 1.5–5% for some large peptides in vitro^7,8^ and 3% for insulin^9^. While recovery certainly depends on catheter properties, such as pore size, membrane length and flow rate^10,11^, it has been speculated that this to some extent is caused by adsorption to the microdialysis membrane and tubings^12,13^. Several other tissue specific factors, including local kinetic properties that control transport of compounds through and from the tissue, may affect how much of a compound is diffused to the microdialysis catheter. Such factors include local blood flow, tissue tortuosity and metabolic activity^10,14–16^.

This study investigates the recovery of insulin from different tissue compartments using microdialysis. We hypothesized that (1) the concentration of insulin decreases from the intravenous compartment to the subcutaneous and intracutaneous tissue, and that (2) adsorption of insulin to the microdialysis membrane and tubing impairs the recovery of insulin from the tissue. The aims of this study were therefore to (1) measure the concentration of insulin in three different compartments (intracutaneous, subcutaneous and intravenous) and (2) to study how much insulin is adsorbed to the microdialysis membranes and tubing during a typical microdialysis experiment, both in vivo and in vitro.

## Methods

### Subjects

Sixteen subjects (seven women) with a mean age of 24 ± 2 years (range 19–28), consecutively recruited through advertising on social media, were included in the study (Table 1). Eleven of these subjects were included to measure insulin concentration in different tissue compartments (2.2) and for analyzing insulin adsorbed to the microdialysis membrane in vivo (2.3.1) (25 ± 1 years). For the in vitro experiments (2.3.2 and 2.4), five subjects were included (21 ± 2 years). All subjects were healthy non-smokers and used no regular medication, except oral contraceptives. On the day of the experiment the subjects arrived in the morning after an overnight fast. They were only allowed to drink water during the experiment. All subjects gave their written consent before participation.

**Table 1:** Demographics of the subjects enrolled in the study.

	Experimental subjects
N	16
Sex (female/male)	7/9
Age (years)	23.9 (2.3)
BMI (kg/m^2^)	22.5 (1.5)
**Blood pressure (mmHg)**	
Before experiment	117/72 (9/8)
After experiment	114/66 (13/8)
**Serum insulin (mU/L)**	
Fasting	5.8 (1.6)
2 h after oral glucose	33.0 (24.7)
**Plasma glucose (mmol/L)**	
Fasting	5.1 (0.3)
2 h after oral glucose	5.8 (1.5)
**Capillary glucose (mmol/L)**	
Fasting	4.6 (0.3)
30 min after oral glucose	7.6 (0.9)
1 h after oral glucose	7.2 (1.4)
2 h after oral glucose	5.8 (1.4)
3 h after oral glucose	4.6 (1.2)
4 h after oral glucose	4.4 (0.4)
HOMA IR	1.3 (0.4)
**Skin temperature (°C)**	
Fasting	30.6 (2.0)
30 min after oral glucose	30.4 (3.3)
1 h after oral glucose	30.9 (3.7)
2 h after oral glucose	31.5 (2.1)
3 h after oral glucose	31.7 (2.1)
4 h after oral glucose	31.4 (1.9)

Data presented in mean (SD).

The study was carried out according to the Helsinki declaration and was approved by the regional ethical review board of Linköping (application ID: DNR 2011/362–31 and DNR 2016/122–32).

### Insulin concentration in different tissue compartments during oral glucose load

In eleven subjects, microdialysis catheters (CMA 71, 10 mm membrane, 100 kDa cut off, M dialysis AB, Stockholm, Sweden) were inserted intracutaneously in the volar skin of the non-dominant forearm and subcutaneously in the periumbilical adipose tissue. The procedure for catheter insertion has previously been described in detail^2,3^, and the intracutaenous catheter placement have been shown with ultrasound to be at a depth of approximately 0.8 mm depth, on the boundary of dermis and hypodermis^2,3,17^. An additional microdialysis catheter for intravenous use (CMA 67, 10 mm membrane, 20 kDa cut off, M dialysis AB, Stockholm, Sweden) was inserted in a peripheral vein in the cubital fossa. A microinjection pump (CMA 107, CMA AB, Solna, Sweden) was connected to each catheter and perfused with a Ringer’s acetate solution with addition of 2.5% albumin and 30 mmol/L urea (APL AB, Umeå Sweden). In the intravenous perfusate, 25 IE/mL heparin (Heparin LEO, Malmö, Sweden) was added to inhibit clotting, according to manufacturer's recommendations. The flow rate was set to 1.0 µL/min. All catheters were perfused for 90 min before they were inserted in the subjects to ensure they were functioning properly.

**Figure 1:**
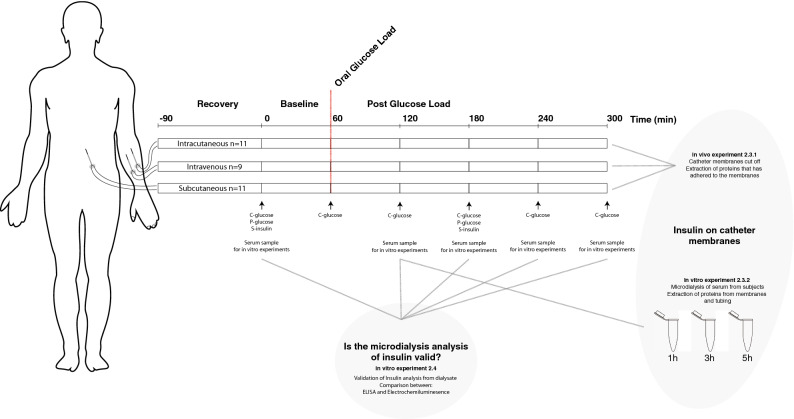
Experimental protocol and setup for in vivo and in vitro experiment in healthy subjects (N = 12). C‐glucose = capillary glucose; P‐glucose = plasma glucose; S‐insulin = serum insulin.

The experimental protocol is shown in Fig. 1. After catheter insertion a 90-min period followed in order to allow the tissue to recover and let the microdialysis system stabilize. Then, a 60-min baseline measurement followed. According to the WHO standard for an oral glucose tolerance test (OGTT), the subjects then ingested 75 g glucose (APL AB, Stockholm, Sweden) diluted in 2 dL water within 5 min to induce an increase of the endogenous insulin concentration. Thereafter four hours of sampling followed to observe the effects of the ingested glucose. Dialysate was sampled in microvials (M dialysis AB, Stockholm, Sweden) at 15-min intervals. Lag time, the time it takes for the microdialysate to flow from the membrane to the microvial, was 5.1 min. To compensate for this, the oral glucose load was therefore ingested 5 min before the exchange of the last vial during baseline. Hence, no compensation for lag time was needed during data analysis. All vials were weighed using a scale (CPA225D, Sartorius Weighing Technology GmbH, Goettingen, Germany) before and after sampling, to determine the volume of the recovered dialysate in each vial.

The dialysate was analyzed for glucose, lactate, pyruvate and urea directly after sampling using ISCUSflex Microdialysis Analyzer (M dialysis, Stockholm, Sweden). Before this analysis, 10 µL was extracted from each vial and pooled hourly in Eppendorf vials to obtain enough volume for insulin analysis. Insulin concentration was measured using enzyme-linked immunosorbent assay (ultrasensitive ELISA, Mercodia AB, Uppsala, Sweden) according to the standard protocol from the manufacturer, and then analyzed using a Clariostar Monochromator Microplate reader with a 450 nm filter (BMG Labtech, Ortenberg, Germany).

During the whole experiment, skin temperature was measured on the surface of the volar part of the same forearm that the intracutaneous and intravenous catheters were inserted in, using a thermometer equipped with a K Type thermocouple (TES 1300, TES Electrical Electronic Corporation, Taipei, Taiwan). Capillary glucose was collected six times during the experiment and analyzed directly using a handheld spectrophotometer (Accu Chek Inform II, Cobas, Switzerland), calibrated according to manufacturer’s recommendations. Venous blood samples were collected during fasting and 2 h after the ingestion of the oral glucose load and analyzed for insulin (using electrochemiluminescence) and glucose (using the hexokinase enzymatic method). The HOMA model (HOMA; [fasting insulin (µU/mL) × fasting glucose (mmol/L)/22.5]) was used to quantify insulin resistance^18^.

At the end of the experiments, catheters were gently removed from the different target tissues. The tip of the catheters, i.e. the catheter membrane and a few millimeters of the tubing, was dipped in natrium chloride to remove red blood cells hampering the analysis and then cut from the microdialysis tubing and stored in an Eppendorf vial at − 86 °C awaiting further analysis.

### Adsorption of insulin to membranes and tubes

#### In vivo catheters

Catheter membranes from seven of the eleven subjects were analyzed for insulin residue (Fig. 1). The frozen membranes were thawed at room temperature. 50 µL phosphate buffered saline (PBS) (Medicago AB, Uppsala, Sweden) with 0.05% Tween 20 (Bio-Rad, California, USA) was added to each vial. After an incubation overnight at 4 °C, the vials were placed on a plate shaker with occasional mixing at room temperature for four hours to extract the adsorbed proteins from the membranes. This method has previously been used for detection of proteins adsorbed to the catheter membrane in surgically treated intracerebral hemorrhage patients^19^. Insulin concentration was then measured using ultrasensitive ELISA (Mercodia AB, Uppsala, Sweden) and a Clariostar Monochromator Microplate reader with a 450 nm filter (BMG Labtech, Ortenberg, Germany).

#### In vitro catheters

Insulin was analyzed directly from the blood sample collected one hour after the oral glucose intake, for reference, using electrochemiluminescence (Fig. 1). Remaining blood was centrifuged in 1800×*g* for 10 min to provide a serum sample which was transferred to microcentrifuge tubes (Ratiolab, 2.0 ml with cap, PP, natural, Dreieich, Germany). The tubes were then placed in a controlled water bath at 37 °C and the serum samples microdialysed using three microdialysis catheters (CMA71, 10 mm, 100 kDa cut off, M dialysis, Stockholm, Sweden). Flow rate and perfusate were identical to as in the in vivo experiments. Vials were exchanged every 60 min. The catheters were removed from the serum bath sequentially, at intervals of one hour, three hours and five hours of sampling. To control for evaporation or depletion of insulin in the microcentrifuge tube, control samples were collected from the serum at catheter removals, respectively. All samples were analyzed using ultrasensitive ELISA.

After sampling, (1) the membranes, (2) a few millimeters of the microdialysis tubing submerged in serum and (3) a few millimeters of the microdialysis tubing not submerged in serum, were cut into small pieces and then placed in separate microcentrifuge tubes (Costar, 1.7 ml with cap, natural, NY, USA). The membranes and tubings were then analyzed using the same protein extraction protocol described in part 2.3.1 and finally analyzed for insulin concentration using ultrasensitive ELISA.

### Validation of ultrasensitive ELISA for detecting insulin in microdialysate

In seven subjects blood samples were also collected during fasting and then hourly after the oral glucose intake in order to compare insulin concentration measured with electrochemiluminescence and ultrasensitive ELISA. Blood samples were analyzed directly, for reference, using electrochemiluminescence, while the remaining blood was centrifuged (1800 g for 10 min) providing serum samples for further in vitro microdialysis experiment (Fig. 1). Experimental setup was identical to 2.3.2 considering controlled water bath, catheters, perfusate and flow rate. Five repeated samplings of 40 min were performed in each serum sample. Control samples from the serum sample, extracted at each vial change, and serum dialysate were analyzed using ultrasensitive ELISA.

### Statistical analysis

Baseline was defined as a mean of the first four measurements. Statistical calculations were performed using GraphPad Prism version 8.0 (GraphPad Software, San Diego, California, USA). The alpha level for statistical significance was set to 0.05.

Paired Student’s t tests were used to compare blood pressure before and after the experiment and differences in volume recovery between the catheters. For analysis of changes in glucose, lactate, pyruvate, urea and insulin in the dialysate (1) between the catheters from the different compartments and (2) between baseline and different time points during the experiment, two-way analysis of variance (ANOVA) for repeated measures followed by multiple comparisons using Sidak’s correction, was used. Changes in skin temperature during the experiment and comparison between insulin adsorbed to the microdialysis membranes used in vivo was analyzed using one-way analysis of variance for repeated measures.

Regression analysis was performed on the data of insulin concentration measured in serum using different methods (electrochemiluminescence and ELISA) described in part 2.4. No statistical analyzes were performed on the data from the in vitro microdialysis experiments described in part 2.3.2 since only two test subjects were included.

#### Missing data

Two intravenous catheters were never inserted because of technical issues during insertion. Due to technical issues during the microdialysate analysis there were some missing values of glucose, lactate, pyruvate and urea. Data from these subjects had to be excluded for the two-way ANOVA tests. The graphs (Figs. 3, 4) are however based on data from all subjects.

When analyzing insulin concentration with ELISA, all analysis results under the detection limit (0.15 mU/L) were set to zero. Insulin concentration from dialysate and membranes of four of the eleven test subjects were unfortunately forced to be excluded due to technical issues during ELISA analysis.

## Results

### Subjects

All subjects were normotensive. No difference in systolic blood pressure was observed during the experiment (*p* = 0.14), however diastolic blood pressure was significantly lower at the end of the experiment compared to the beginning of the experiment (*p* = 0.005). Mean skin temperature (SD) during the experiment was 31.0 (0.5)°C. No significant change in skin temperature was observed during the experiment (*p* = 0.69). Neither the HOMA model nor plasma insulin and glucose levels indicated that any of the subjects were insulin resistant (Table 1). Detailed numeric data can be found in Supplementary files.

### Volume recovery

Mean (SD) volume recovery in the intravenous catheters was 99 ± 2%, which was significantly higher (*p* = 0.006) than in the intracutaneous (96 ± 1%) and subcutaneous catheters (96 ± 1%).

### Insulin concentration in different tissue compartments during oral glucose load

#### Dialysate insulin concentration

**Figure 2:**
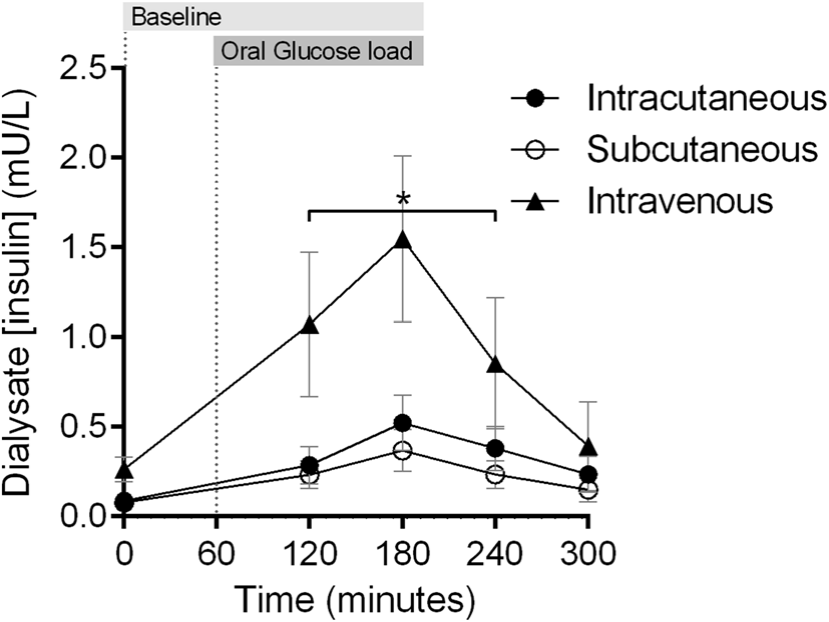
Mean change in dialysate concentration of insulin in the intracutaneous, subcutaneous and intravenous compartment after oral intake of 75 g glucose load in healthy subjects (N = 8). *Indicates a significant difference between the intravenous catheter compared to the intra- and subcutaneous catheters. Error bars represent standard error of the mean (SEM).

The concentration of insulin in the dialysate increased significantly in the intravenous compartment (*p* = 0.033), but not in the intracutaneous (*p* = 0.23) or subcutaneous (*p* = 0.53) compartments (Fig. 2), after the oral glucose load. The peak insulin concentration was significantly higher in the intravenous compartment compared to the other compartments (*p* = 0.047). No difference was observed in insulin concentration between the intracutaneous and subcutaneous compartments (*p* = 0.95).

#### Metabolic markers in vivo

**Figure 3:**
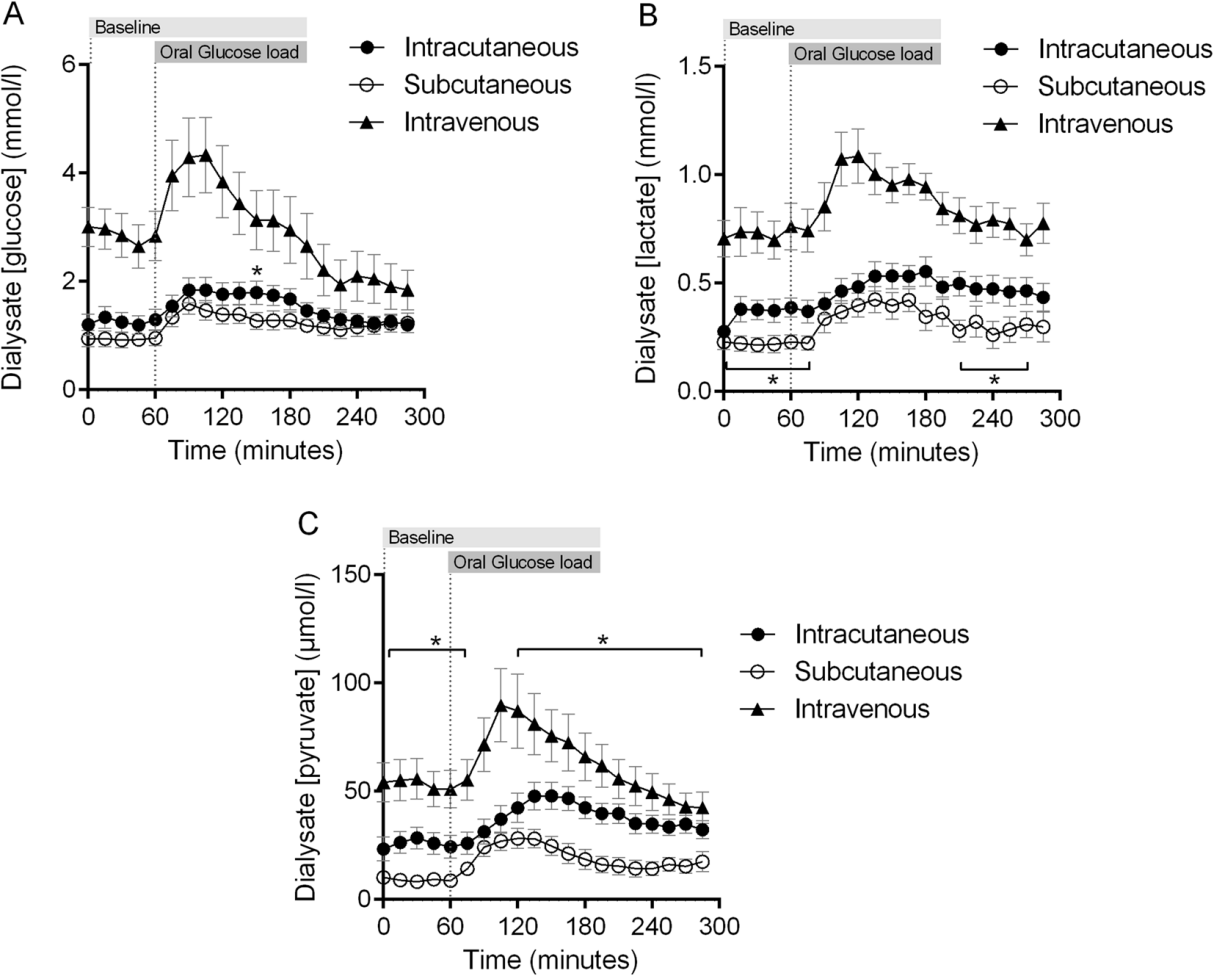
Mean change in dialysate concentration of glucose, lactate and pyruvate in the intracutaneous, subcutaneous and intravenous compartment after oral intake of 75 g glucose load in healthy subjects (N = 11). The concentration of glucose, lactate and pyruvate were all significantly higher in the intravenous catheter than the intra- and subcutaneous catheters during the whole experiment, with one exception for pyruvate 270 min into the experiment. *Indicates a significant difference between the intra- and subcutaneous catheters. Error bars represent standard error of the mean (SEM).

An increase in all metabolic markers (glucose, lactate and pyruvate) was observed in the dialysate after the oral glucose load, in all three catheters (Fig. 3). During the whole experiment, the concentrations of glucose, lactate and pyruvate were significantly higher in the intravenous catheter compared to the intracutaneous and subcutaneous catheters (*p* = 0.03), except from a single time point in the end of the experiment (T = 270 min) where the difference between the intracutaneous and the intravenous catheter was non-significant for pyruvate (*p* = 0.09). The concentration of all the metabolic markers was generally higher in the intracutaneous catheter compared to the subcutaneous catheter during the whole experiment. Significant differences are highlighted in the graphs with asterisks.

#### Urea in vivo

**Figure 4:**
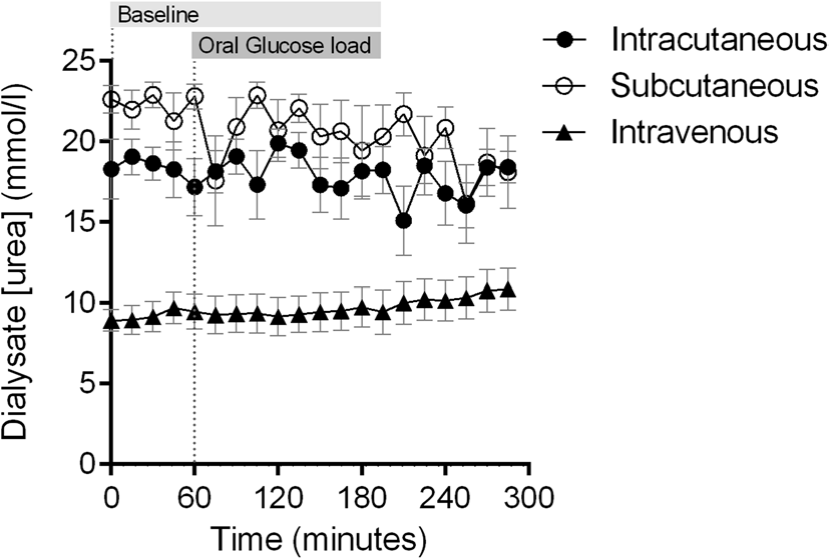
Mean changes in dialysate concentration of urea in the intracutaneous, subcutaneous and intravenous compartment after oral intake of 75 g glucose load in healthy subjects (N = 11). *Indicates a significant difference between the intra- and subcutaneous catheters. Error bars represent standard error of the mean (SEM).

The concentration of urea was stable in all three compartments during the whole experiment (Fig. 4). No change in urea concentration compared to baseline was observed in any of the three catheters (*p* = 0.34). The absolute recovered concentration of urea was however markedly lower in the intravenous catheter than the intracutaneous and subcutaneous catheters (*p* = 0.014), indicating higher blood flow surrounding the intravenous catheter compared to the other two catheters.

### Adsorption of insulin to membranes and tubes

#### In vivo

In Table 2, the concentration of insulin extracted from the membranes in the different compartments is presented. The adsorption of insulin to the microdialysis membrane was highest in the intravenous compartment (*p* = 0.01). No difference was observed in adsorption of insulin between the intracutaneous and subcutaneous compartments (*p* = 1.0).

**Table 2:** Concentration of insulin adsorbed to the microdialysis membranes from the different in vivo compartments (intracutaneous, subcutaneous and intravenous), analyzed with ELISA.

Catheter compartment	Mean [insulin] mU/L (SD)
Intracutaneous	0.22 (0.24)
Subcutaneous	0.23 (0.14)
Intravenous	0.99 (0.39)

Data presented in mean (SD).

#### In vitro

Insulin could be detected on both the microdialysis membranes and tubings (Table 3), however the adsorption to the tubings was markedly lower compared to the adsorption to the membranes (membrane:tubing descended in serum 4:1, membrane: tubing not descended in serum 6:1). While the insulin concentration in the dialysate was relatively stable over time, the adsorption to the membrane was time dependent, with the most adsorption happening during the first hour, i.e. on the membrane removed after one hour of microdialysis sampling. The quota of adsorption of insulin to the membrane and tubings in relation to insulin concentration in the dialysate did also decrease over time. The relative recovery of insulin varied between 30.2% and 50.8% and tended to increase over time (not significant).

**Table 3:** Concentration of insulin (mU/L) adsorbed to the microdialysis membrane (insulin_m_), tubing descended in serum (insulin_ts_), tubing not descended in serum (insulin_t_), dialysate (insulin_d_) and control samples from the serum bath after sampling (insulin_s_) in vitro.

Subject	Sampling time (h)	[insulin_m_]	[insulin_ts_]	[insulin_t_]	[insulin_s_]	[insulin_d_]	[insulin_m_]/[insulin_s_]	[insulin_m_]/[insulin_d_]	[insulin_ts_]/[insulin_d_]	[insulin_t_]/[insulin_d_]	RR [insulin_d_]/[insulin_s_]
1	1	5.7	0.91	0.37	46.8	18.4	12.2%	31.0%	5.0%	2.0%	39.4%
1	3	1.8	0.60	0.37	53.4	16.1	3.3%	11.2%	3.7%	2.3%	30.2%
1	5	1.1	0.14	0.15	36.5	17.3	2.9%	6.4%	0.8%	0.9%	47.5%
2	1	0.96	0.32	0.26	11.4	4.6	8.4%	20.9%	7.0%	5.7%	40.5%
2	3	0.36	0.18	0.19	10.4	5.3	3.4%	6.8%	3.4%	3.6%	50.8%
2	5	0.28	0.37	0.24	9.6	4.4	2.9%	6.4%	8.4%	5.5%	45.8%

RR = relative recovery. Insulin concentration analyzed using ELISA.

### Validation of ELISA for assessment of insulin in microdialysis

**Figure 5:**
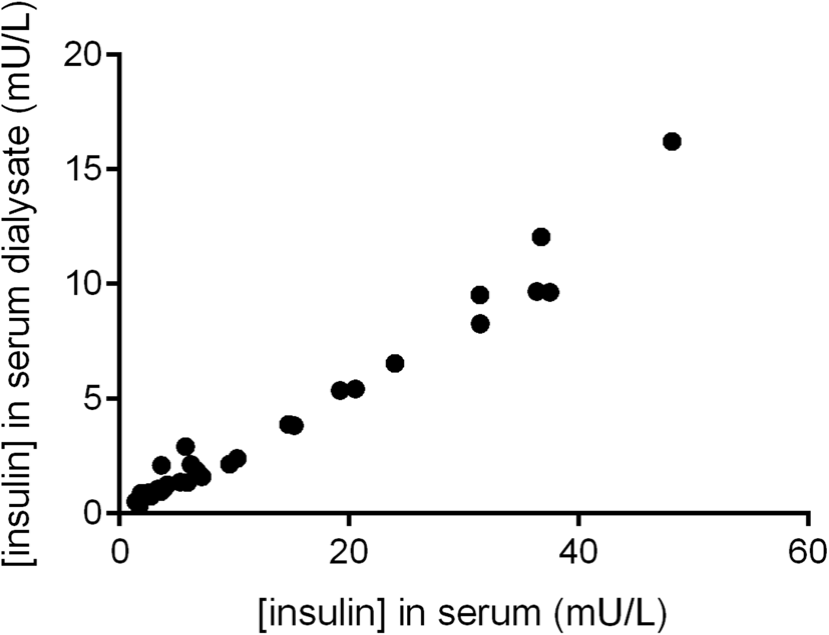
Correlation between insulin concentration in serum and insulin concentration in serum dialysate, measured with ELISA.

Linear regression of insulin concentration in serum measured with electrochemiluminescence compared to ultrasensitive ELISA showed a slope value of 0.70 and R^2^ of 0.92. Insulin concentration measured with ultrasensitive ELISA directly in serum was correlated to the insulin concentration in the microdialysate analyzed with ultrasensitive ELISA using linear regression (Fig. 5), with a slope value of 0.30 and R^2^ of 0.97.

## Discussion

In this paper we show that the concentrations of insulin extracted by microdialysis intravenously, in subcutaneous tissue and in the skin after an oral glucose load are different, and that the measured concentrations are affected by adsorption to the membrane.

The measured insulin concentration in the dialysate obtained from intravenous catheters is significantly higher than in intracutaneous and subcutaneous catheters, indicating that there is a decrease in tissue concentration of insulin in more peripheral tissue compartments. Even though we did not find a significant difference in concentration between the concentration of insulin in catheters placed in intracutaneous and subcutaneous tissue, there seems to be a tendency towards lower insulin concentrations in tissues with decreasing vascularity.

In this paper we also demonstrate how insulin is adsorbed to the microdialysis membrane, both after in vivo microdialysis after an oral glucose load, but also in in vitro experiments on serum samples after the same glucose provocation. The amount of insulin adsorbed to the microdialysis membrane was highest for catheters that had been placed intravenously in healthy subjects (in vivo). This was expected, since the concentration of insulin also was greater intravenously. The adsorption of insulin to the membrane of catheters that were placed in a serum bath (in vitro) seemed to be time dependent, with a rapid initial increase that subsided over time. This time-dependency suggests that insulin may be adsorbed rapidly to the membrane, after which it gradually detaches from the membrane after the first hour of microdialysis sampling. It may be speculated that the adsorption profile of insulin to the membrane may affect the relative recovery. In our study we saw a tendency towards better relative recovery over time, however this was not statistically tested due to the low number of samples (Table 3). It is known that the insertion of microdialysis catheters cause a local trauma in the tissue, and that a recovery time for the tissue is necessary for that matter^20,21^. The time dependent adsorption of insulin to the catheter after insertion, which we observed in our study, indicates that there may be a need to not only let the tissue adapt to the catheter insertion, but also to let the microdialysis system have time to stabilize and adapt before dialysate analysis is done. An elegant and interesting addition to these results would have been to investigate the time/adsorption correlation in an in vivo experiment. As the concentration of insulin is dynamic in vivo this would however be challenging. It would also require insertion of numerous catheters in each subject, which ethically would be questionable.

Insulin is known to be adsorbed by different materials such as glass and polyacrylamide^22^. Relatively few studies have investigated adsorption to the microdialysis membrane (polyarylethersulphone). In Stensson et al.^23^ investigated the specific adsorption of endocannabinoids to the microdialysis membrane in vivo. Rather than having a static accumulation rate over time, a dynamic adsorption pattern of endocannabinoids to the membrane in muscle and skin was observed. Similar to our study did the adsorption occur rapidly initially and relative adsorption decreased over time. The author’s speculated whether the decrease in relative recovery could be due to tissue depletion of the analytes of interest^23^. No signs of depletion of insulin was however observed in this study (Table 3). One could speculate, but not confirm, if the "stickiness" of the molecules to the membranes decrease over time in the tissue or tissue bath.

Different microdialysis catheter modifications with the aim to decrease adsorption of different analytes to the microdialysis membrane have been explored. Yoshida et al. siliconized the microdialysis tubing to avoid adsorption of hypocretins^24^. Dahlin et al. coated the microdialysis membrane with the triblock copolymer Pluronic F-127^13,25^ and Poloxamer 407^26^ to reduce protein adsorption and increase fluid recovery, the latter with a 33% decrease of protein adsorption^26^. How coating affects the adsorption of insulin has however not been studied, but could be interesting to investigate in future studies.

Electrochemiluminescence is traditionally the golden standard to measure insulin concentration, however ultrasensitive ELISA is more commonly used in microdialysis studies because the required volume of the samples is much lower. To make sure that the low intracutaneous concentrations of insulin are not caused by a low sensitivity of the ELISA technique, we compared the two methods in vitro. Linear regression analysis shows that ultrasensitive ELISA underestimate the insulin concentration compared to electrochemiluminescence (slope value of 0.70). This indicates that the assessment of insulin, in all microdialysis experiments from all tissue compartments, are undervalued by approximately 30%, if ELISA is used for the measurement. Further analysis of this is however needed before an accurate conversion value can be established. This should be considered when comparing serum values analyzed by electrochemiluminescence with dialysate concentrations from ELISA.

Difficulties with fluid recovery due to fluid loss to the surrounding medium or tissue is a well-known issue during microdialysis experiments. Interestingly, despite adsorption of insulin and other molecules that might affect the pore size and the properties of the microdialysis membrane, fluid recovery was close to 100% in our current study. In combination with adequate recovery of metabolites (glucose, lactate and pyruvate) in the dialysate, we therefore do not think that the low recovery of insulin from the peripheral compartments is not caused by imbalance in filtration over the microdialysis membrane.

Despite the fact that insulin levels are lower in peripheral tissues, such as the skin, our results indicate that it has an active local role in maintaining local metabolic homeostasis as well as having an effect on the local microcirculation. Previous studies using microdialysis in the skin, for example in burn patients^27^ and in reconstructive surgery^28^, show local effects in the skin that may not be seen in the central circulation, suggesting an un-coupling effect between local tissue homeostasis and central vital functions. Therefore, a better understanding of the connection between local impaired glucose metabolism and ischemia is essential to finding ways to prevent morbidity, and the need for further surgery when the skin homeostasis is challenged.

### Limitations

This study has several limitations. One limitation is that we do not know the exact intracutaneous and subcutaneous concentrations of insulin in the respective tissue compartment in vivo. We can therefore only make assumptions on the recovery of the system, mainly based on data from in vitro experiments, and are therefore unable to calculate the absolute recovery for insulin in the tissue. The conclusions of the paper are therefore confined to assessment of differences in tissue concentration between the different compartments. Hypothetically, tissue biopsies could provide accurate insulin concentrations from each compartment for reference.

A second limitation is that we only have investigated the adsorption of insulin to the microdialysis membrane in vitro and not in vivo. As the concentration of insulin is dynamic in vivo, such measurements would be challenging. It would also require insertion of numerous catheters in each subject, which ethically would be questionable.

Finally, the catheter cut-off is different in the intracutaneous and subcutaneous catheters (100 kDa) than in the intravenous catheters (20 kDa). The significance is however regarded minor, as the molecular weight of the metabolic active insulin (5.8 kDa) is fairly below 20 kDa.

## Conclusion

This study concludes that (1) the concentration of insulin in peripheral tissues (intracutaneous and subcutaneous) as measured using microdialysis is low, which possibly is related to decreasing tissue vascularity and blood flow, and (2) adsorption of insulin to the microdialysis membrane does not seem to hamper the recovery of insulin as much as previously suspected. However, it seems to be a time dependent correlation of adsorption, implying that it is important not only to let the tissue recover after the catheter insertion, but also to let the microdialysis system stabilize before sampling begins.

Furthermore, the fact that adsorption to the membrane occurs, particularly during the first hour of microdialysis, highlights the importance of including adsorbed large peptides trapped in the membrane when interpreting the microdialysate data of the target tissue.

## Supplementary Information

Supplementary Information.

Supplementary Information.

## Abbreviations

ELISA: Enzyme-linked immunosorbent assay
HOMA: Homeostatic model assessment
OGTT: Oral glucose tolerance test
SEM: Standard error of the mean
SD: Standard deviation
