# Supplementary material for: Sampling insulin in different tissue compartments using microdialysis: methodological aspects
Source: Sci Rep. 2020 Dec 15;10:21948. doi: 10.1038/s41598-020-78728-x (PMC7738523; doi:10.1038/s41598-020-78728-x)
Supplement: Supplementary file 1 — Supplementary Information. [file 41598_2020_78728_MOESM1_ESM.docx]

Supplementary information

Sampling insulin in different tissue compartments using microdialysis: methodological aspects

Alexandra Högstedt*^1^, Bijar Ghafouri^2^, Erik Tesselaar^3^, Simon Farnebo^4^

1. Department of Surgery in Linköping, and Department of Biomedical and Clinical Sciences, Linköping University, Linköping, Sweden
2. Pain and Rehabilitation Centre, and Department of Health, Medicine and Caring Sciences, Linköping University, Linköping, Sweden
3. Department of Medical Radiation Physics, and Department of Health, Medicine and Caring Sciences, Linköping University, Linköping, Sweden
4. Department of Hand Surgery, Plastic Surgery and Burns, and Department of Biomedical and Clinical Sciences, Linköping University, Linköping, Sweden
